# A new promising way of maintenance therapy in advanced ovarian cancer: a comparative clinical study

**DOI:** 10.1186/s12885-018-4792-9

**Published:** 2018-09-20

**Authors:** Vsevolod I. Kiselev, Levon A. Ashrafyan, Ekaterina L. Muyzhnek, Evgeniya V. Gerfanova, Irina B. Antonova, Olga I. Aleshikova, Fazlul H. Sarkar

**Affiliations:** 1 Peoples’ Friendship University of Russia, Moscow, Russian Federation; 2 Russian Scientific Center of Roentgenoradiology, Moscow, Russian Federation; 3 MiraxBioPharma, Joint-Stock Company, Valovaya Ul., 21, build. 125, Moscow, Russian Federation 115054; 4 Department of Pathology, Wayne State University (Retired as Distinguished Professor), Detroit, MI USA

**Keywords:** Ovarian cancer, Survival, Maintenance therapy, Indole-3-carbinol, Epigallocatechin-3-gallate

## Abstract

**Background:**

There is an urgent need for more novel and efficacious therapeutic agents and strategies for the treatment of ovarian cancer - one of the most formidable female malignancies. These approaches should be based on comprehensive understanding of the pathobiology of this cancer and focused on decreasing its recurrence and metastasis. The aim of this study was to evaluate the efficacy of five-year maintenance therapy with indole-3-carbinol (I3C) as well as I3C and epigallocatechin-3-gallate (EGCG) conducted before, during, and after combined treatment compared with combined treatment alone in advanced ovarian cancer.

**Methods:**

Patients with stage III-IV serous ovarian cancer were assigned to receive combined treatment plus I3C (arm 1), combined treatment plus I3C and EGCG (arm 2), combined treatment plus I3C and EGCG plus long-term platinum-taxane chemotherapy (arm 3), combined treatment alone without neoadjuvant platinum-taxane chemotherapy (control arm 4), and combined treatment alone (control arm 5). Combined treatment included neoadjuvant platinum-taxane chemotherapy, surgery, and adjuvant platinum-taxane chemotherapy. The primary endpoint was overall survival (OS). Secondary endpoints were progression-free survival (PFS) and rate of patients with recurrent ovarian cancer with ascites after combined treatment.

**Results:**

After five years of follow-up, maintenance therapy dramatically prolonged PFS and OS compared to control. Median OS was 60.0 months (95% CI: 58.0–60.0 months) in arm 1, 60.0 months (95% CI: 60.0–60.0 months) in arms 2 and 3 while 46.0 months (95% СI: 28.0–60.0 months) in arm 4, and 44.0 months (95% СI: 33.0–58.0 months) in arm 5. Median PFS was 39.5 months (95% СI: 28.0–49.0 months) in arm 1, 42.5 months (95% СI: 38.0–49.0 months) in arm 2, 48.5 months (95% СI: 39.0–53.0 months) in arm 3, 24.5 months (95% СI: 14.0–34.0 months) in arm 4, 22.0 months (95% СI: 15.0–26.0 months) in arm 5. The rate of patients with recurrent ovarian cancer with ascites after combined treatment was significantly less in maintenance therapy arms compared to control.

**Conclusions:**

Long-term usage of I3C and EGCG may represent a new promising way of maintenance therapy in advanced ovarian cancer patients, which achieved better treatment outcomes.

**Trial registration:**

Retrospectively registered with ANZCTR number: ACTRN12616000394448. Date of registration: 24/03/2016.

## Background

Ovarian cancer (OC) has long been one of the most difficult and treacherous female cancers, accounting for nearly 150,000 lethal cases annually worldwide [1]. Various estimates put five-year overall survival with advanced OC at 12–42%. Maximal cytoreductive surgery, followed by platinum-taxane chemotherapy (TP and TC regimens) has been standard treatment of OC since 1996. However, 60–80% of such patients relapse in six to 24 months, which requires further chemotherapy and eventually makes the tumor chemoresistant. The relapse with chemoresistant tumors results in grievous complications (ileus, ascites, cachexia) leading to early death.

More effective OC treatment strategies are urgently required to improve survival. They should obviously be focused on minimizing recurrence rate and metastasis and overcoming drug resistance. Accumulating evidence suggests that it cannot be done solely with special regimens of standard chemotherapy [2–4], or a combination of conventional chemotherapy and monotargeted antitumor drugs [5–8]. Targeted antitumor drugs used in a maintenance therapy regimen have recently gained increasing attention as a promising management option for recurrent OC, helping to extend progression-free intervals [9–13].

Since the mid-1990s, a new theory behind the nature of cancer has been steadily gaining traction, namely that of a dominating role of cancer stem cells (CSCs), also known as ‘cancer-initiating cells’ or ‘tumor-initiating cells,’ a special rare population of immortal aggressive tumorigenic cells capable of self-renewal and pluripotency. There is a huge array of evidence suggesting that CSCs resistant to conventional chemo- and radiotherapy are responsible for cancer initiation, progression, metastasis, and recurrence, as well as radio- and drug resistance [14–16]. Superior CSCs resistance to anti-cancer drugs is explained biologically by their hyperexpression of multidrug efflux transporters, antiapoptotic factors, and DNA repair and detoxifying enzymes [16–18].

The concept of cancer stem cells has been proven both experimentally and clinically in many cancers, including OC [19, 20]. Ovarian CSCs were confirmed to play a big role in the development of chemoresistance and generation of recurrent and metastatic foci in OC [21, 22].

Search for and development of drugs inhibiting CSCs is a new unfolding opportunity for targeted antitumor therapy, which can affect the current paradigm of anti-cancer drug development in general. Over the last decade, a lot of work has been done to develop new drugs to target CSCs. There are four known groups of molecular targets for anti-CSCs therapy: 1) cell-surface molecular markers of CSCs; 2) proteins of various signaling pathways, controlling CSCs survivability and differentiation; 3) membrane transporters crucial for CSCs anticancer multidrug resistance, and 4) CSCs cellular microenvironment (“niche”) molecular factors [16, 17]. Some of the newly identified compounds that selectively target CSCs have been evaluated in preclinical and clinical studies [17]. Within the broader context of improving the overall suffering and survival of oncological patients, such selective CSCs inhibitors are suggested for concomitant use with conventional chemotherapeutic drugs whose important role is to eliminate bulk tumor cells [23].

Our сomparative clinical trial investigated the efficacy of long-term maintenance therapy with indole-3-carbinol (I3C) as well as maintenance therapy with I3C and epigallocatechin-3-gallate (EGCG), agents demonstrating multiple antitumor activities, including specifically the inhibition of CSCs. The current study enrolled untreated advanced OC patients.

## Methods

### Patient population

All eligible women were ≥ 39 years of age with histologically confirmed International Federation of Gynecology and Obstetrics (FIGO) stage III to IV serous epithelial OC (serous carcinoma) defined as high-grade (Grade III) serous carcinoma according to the WHO grading system [24].

Eligible patients also met the following criteria: Eastern Cooperative Oncology Group (ECOG) performance status ≤ 2; adequate hematologic, hepatic, and renal functions: absolute granulocyte count ≥ 1.5 × 10^3^/mm^3^; platelets ≥ 100 × 10^3^/mm^3^; bilirubin, creatinine within normal limits; aspartate aminotransferase (AST) and alanine aminotransferase (ALT) < 2.0 times normal upper limit; AST and ALT < 5.0 times normal upper limit if liver metastases present; absence of significant comorbidities (documented history of gastric/duodenal ulcer or heart attack within the last 12 months; polyneuropathy; decompensated diabetes), and submission of written informed consent.

Ineligibility criteria included *BRCA* genes mutations, malignancies of other localizations, positive RW or HIV tests, alcohol or drug abuse, pregnancy or lactation, logistical issues (remote residence etc.), or any uncontrolled psychiatric illnesses or conditions potentially hampering compliance and/or monitoring, other severe comorbidities potentially (investigator discretion) affecting the patient’s ability to participate in the trial.

All study procedures (the study protocol) were approved by the local Ethics Committee of the Federal State Budgetary Institution “Russian Scientific Center of Roentgenoradiology” (RSCRR) of the Ministry of Healthcare of the Russian Federation and conducted in accordance with the principles of Good Clinical Practice and Declaration of Helsinki. All patients submitted written informed consent at the time of enrollment.

Peritoneal cancer index (PCI) was determined for all patients in the study at screening using data obtained by thoracoabdominal computed tomography to assess the initial tumor spread [25].

All required procedures were carried out by the same surgical team.

### Study design and treatment

To provide maintenance therapy effect estimates, the original plan was to enroll 300 patients, with 60 patients per arm. The target sample size (*n* = 300) was also determined by the number of eligible patients at RSCRR during 5-year period of patient enrollment from January 2004 through December 2009.

According to the initial protocol, patients were to be randomly assigned to receive combined treatment plus I3C continuously (arm 1), combined treatment plus I3C and EGCG continuously (arm 2), combined treatment plus I3C and EGCG continuously plus long-term platinum-taxane chemotherapy (arm 3), combined treatment alone without neoadjuvant chemotherapy (arm 4), and combined treatment alone (arm 5). In the process of enrollment, it turned out to be problematic to get written consent to randomization from enough advanced OC patients to complete five arms balanced by the number of patients. So the decision was made, after discussion with the RSCRR ethics committee, that patients should be enrolled on the basis of treatment preference (patients’ choice). The trial protocol was modified accordingly. As a result, 284 patients were enrolled in January 2004 through December 2009 at RSCRR and treated in accordance with their choice made at the moment of diagnosis (arm 1, *n* = 46; arm 2, *n* = 76; arm 3, *n* = 42; arm 4, *n* = 40; arm 5, *n* = 80). Hofmann MA et al. [26] earlier described a similar enrollment issue in a clinical study of advanced melanoma, with the same solution.

**Fig. 1:**
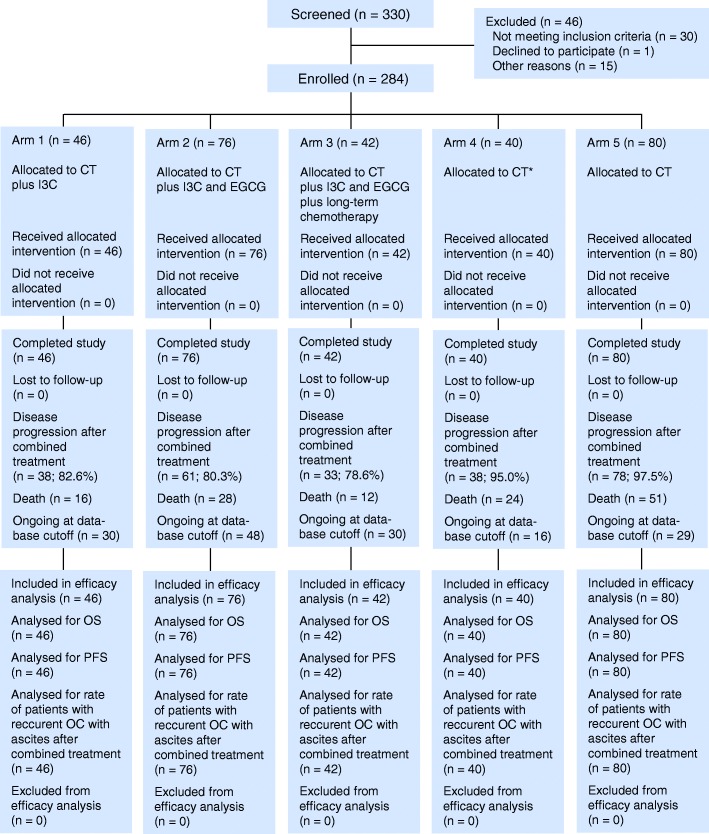
CONSORT diagram. *CT* combined treatment with neoadjuvant chemotherapy; CT* combined treatment without neoadjuvant chemotherapy; *OS* overall survival; *PFS* progression-free survival; *OC* ovarian cancer

According to the modified protocol, all enrolled patients were offered to choose from five treatment options: combined treatment plus twice daily oral administration of 200 mg of I3C continuously (arm 1), combined treatment plus twice daily oral administration of 200 mg of I3C and 200 mg of EGCG continuously (arm 2), combined treatment plus twice daily oral administration of 200 mg of I3C and 200 mg of EGCG continuously plus long-term platinum-taxane chemotherapy, 2–3-month cycles (arm 3), combined treatment alone without neoadjuvant chemotherapy (arm 4), and combined treatment alone (arm 5) (Fig. 1). Combined treatment included neoadjuvant chemotherapy (NACT) consisting of two to four three-week cycles of TP regimen (1st day: intravenous paclitaxel 175 mg/m^2^ with premedication; 2nd day: intravenous cisplatin 75–100 mg/m^2^ with hyperhydration) or TC regimen (1st day: intravenous paclitaxel 175 mg/m^2^ with premedication; 2nd day: intravenous carboplatin AUC 5), primary surgery (panhysterectomy with subtotal resection of the greater omentum and the maximum removal of disseminated tumor foci), carried out 28 days after the last cycle of NACT, and postoperative adjuvant chemotherapy (ACT) consisted of five to six 3-week cycles of TP or TC regimen performed 14 days after surgery. Platinum-taxane chemotherapy ТР and TC regimen were distributed almost evenly in each arm.

Patients in combined treatment arms 1, 2, 3, and 5 had a high perioperative risk profile or a low likelihood of achieving cytoreduction to < 1 cm of residual disease (ideally to no visible disease). Thus, the combined treatment with NACT was conducted in these arms according to generally accepted international treatment guidelines [27, 28] as well as to Russian Federation treatment guidelines and local RSCRR treatment guidelines for advanced OC. In accordance with RSCRR treatment guidelines for FIGO III-IV OC (Protocol № С56/10, order № 80-о dated 17.08.2010), a large volume of ascitic fluid in the abdomen and СА-125 level more than 500 U/ml are additional criteria for unresectability by primary debulking and presurgery NACT. At screening, the rates of patients with ascites in all arms were about 70% and PCI medians were from 24 to 29 (Table 1). It was shown earlier that PCI > 10 was positively associated with a poor prognosis for any intra-abdominal and intrapelvic malignant tumor with peritoneal spread, including advanced OC [25, 29].

**Table 1:** Patient demographic and clinical characteristics

Characteristic	Arm 1 (*n* = 46)	Arm 2 (*n* = 76)	Arm 3 (*n* = 42)	Arm 4 (*n* = 40)	Arm 5 (*n* = 80)
Age, years					
Median	54.0	54.0	54.5	54.2	54.1
Range	40–76	43–71	41–68	47–68	39–69
Ethnicity, No. (%)					
White	42 (91.3)	68 (89.5)	38 (90.5)	36 (90)	73 (91.2)
Asian	4 (8.7)	7 (9.2)	4 (9.5)	3 (7.5)	6 (7.5)
Black	0 (0)	0 (0)	0 (0)	0 (0)	0 (0)
Other	0 (0)	1 (1.3)	0 (0)	1 (2.5)	1 (1.2)
FIGO stage at screening, No. (%)					
III	38 (82.6)	60 (78.9)	34 (80.9)	32 (80.0)	66 (82.5)
IV	8 (17.4)	16 (21.1)	8 (19.0)	8 (20.0)	14 (17.5)
PCI					
Median	24	27	27	27	29
Range	9–36	8–37	7–37	7–37	7–37
7–10 (≤ 10), No. (%)	2 (4.3)	4 (5.3)	4 (9.5)	2 (5.0)	2 (2.5)
11–20	12 (26.1)	20 (26.3)	8 (19.0)	8 (20.0)	20 (25.0)
21–30	23 (50.0)	26 (34.2)	14 (33.3)	17 (42.5)	26 (32.5)
31–37	9 (19.6)	26 (34.2)	16 (38.1)	13 (32.5)	32 (40.0)
ECOG performance status at screening, No. (%)					
0	40 (87.0)	67 (88.2)	37 (88.1)	34 (85.0)	69 (86.3)
1	3 (6.5)	5 (6.6)	3 (7.1)	4 (10.0)	6 (7.5)
2	3 (6.5)	4 (5.3)	2 (4.8)	2 (5.0)	5 (6.3)
*p*^a^	0.96	0.84	0.86	0.93	
Rate of patients with ascites					
at screening^b^, No. (%)	31 (67.4)	51 (67.1)	29 (69.0)	28 (70.0)	55 (68.8)
95% CI	52–80	55–68	53–82	54–83	57–79
*p*^b^	0.87	0.82	0.97	0.89	
Standard chemotherapy regimen, No. (%)					
ТР	26 (57)	40 (53)	20 (48)	18 (45)	37 (46)
ТС	20 (43)	36 (47)	22 (52)	22 (55)	43 (54)
CA-125 level, U/mL					
at screening					
Mean	579.78	584.32	581.85	581.98	583.75
Range	110- > 600	115- > 600	120- > 600	110- > 600	105- > 600
at presurgery					
Mean ± SD^c^	31.50 ± 5.19	37.91 ± 21.43	42.26 ± 24.50	581.98 ± 85.07	68.70 ± 16.23
Range	25–45	30–210	30–190	69- > 600	35–110
after combined treatment					
Mean ± SD^c^	12.78 ± 2.78	10.42 ± 4.07	12.67 ± 5.48	31.05 ± 8.70	32.44 ± 6.23
Range	10–20	8–42	8–35	20–54	20–55
Primary debulking surgery at combined treatment^d^, No. (%)					
Complete cytoreduction (no visible tumor foci)	39 (84.8)	63 (82.9)	34 (81.0)	5 (12.5)	20 (25.0)
Optimal cytoreduction (≤ 1 cm)	7 (15.2)	13 (17.1)	8 (19.0)	21 (52.5)	51 (63.8)
Suboptimal cytoreduction (> 1 cm)	0 (0)	0 (0)	0 (0)	14 (35.0)	9 (11.3)
Disease progression (tumor recurrence rate) after combined treatment within five years of follow-up, No. (%)	38 (82.6)	61 (80.3)	33 (78.6)	38 (95.0)	78 (97.5)
Rate of patients without recurrent ovarian cancer within five years of follow-up, No. (%)	8 (17.4)	15 (19.7)	9 (21.4)	2 (5.0)	2 (2.5)

*FIGO* International Federation of Gynecology and Obstetrics, *PCI* peritoneal cancer index, *ECOG* Eastern Cooperative Oncology Group, *95% CI* 95% confidence interval, *SD* standard deviation

^a^Mann-Whitney U-test was applied to determine the differences between arms 1–4 vs arm 5

^b^Chi-square criterion was applied to determine the differences between arms 1–4 vs arm 5

^c^Student’s test was applied to determine mean level, standard deviation, and the differences between arms 1–3 vs arm 5

All differences between arms 1–3 vs arm 5 were statistically significant (*p* <  0.0001)

^d^Mann-Whitney U-test was applied to compare the degree of surgery used in arms 1–3 vs arm 5. Degrees of surgery were scored as follows: macroscopic completed resection (no visible tumor foci) – 0, optimal debulking (≤ 1 cm) – 1, suboptimal debulking (> 1 сm) – 2. All differences between arms 1–3 vs arm 5 were statistically significant (*p* <  0.0001)

The efficacy of NACT was evaluated by CA-125 level dynamics according to the Rustin criterion [30] and by tumor response per RECIST criteria [31]. Clinical manifestations of NACT success were the disappearance of ascites, reduction of tumor foci size, their smaller dissemination that allowed to perform the subsequent surgical operation as completely as possible and to reduce the risk of postoperative complications. The number of NACT cycles (from two to four) depended on time to CA-125 ≤ 35 U/mL, general declining profile of CA-125, general condition, and laboratory and diagnostic data.

The efficacy of ACT was estimated per RECIST criteria [31] in 21 days after the end of the last ACT cycle. The number of ACT cycles (five or six) depended on personal clinical and laboratory characteristics of every patient, namely, residual tumor size, CA-125 level, general condition, and laboratory and diagnostic data.

Maintenance therapy with I3C as well as maintenance therapy with I3C and EGCG started 14 days prior to combined treatment and continued through combined treatment and for 5 years of the follow-up period.

I3С is the active substance of medical drug Indinol^®^Forto, capsules, 200 mg I3C per capsule (MiraxBioPharma, Joint-Stock Company, Russia) [32]. I3C (100 mg per capsule) and EGCG (100 mg per capsule) are the active components of dietary supplement Promisan^®^ (MiraxBioPharma, Joint-Stock Company, Russia) [33].

If the disease progressed (growth of existing or verification of new OC foci), patients were recommended to undergo chemotherapy as per approved protocol, depending on the length of the platinum-free interval (date of last platinum administration to date of progression). If an OC patient relapsed but background factors were beneficial (no ascites, complete cytoreductive surgery, long platinum-free interval, and general satisfactory status), a possibility for secondary debulking surgery was considered. Patients for secondary debulking surgery were selected on the basis of the AGO score developed and validated in DESKTOP I/II trials [34, 35].

### Study endpoints

The primary endpoint was overall survival (OS) defined as the interval between the date of diagnosis and the date of death from any cause. The first secondary endpoint was progression-free survival (PFS) defined as time from random assignment to disease progression per RECIST, clinical progression (per investigator) or CA-125 progression (per GCIG criteria), or death from any cause. The second secondary endpoint was the rate of patients with recurrent OC with ascites after combined treatment within 5 years of follow-up.

### Efficacy and toxicity assessment

Primary efficacy analyses included all the intent-to-treat patients. Positron emission tomography-computed tomography (PET-CT) or magnetic resonance imaging (MRI) was performed at baseline and every 3 months throughout the study period. Ultrasonography was performed every month during year 1, and then at least every 3 to 4 months during years 2–3 and every 6 months during years 4–5. CA-125 test was performed by a local laboratory monthly during the study.

Disease recurrence was defined as an objective clinical diagnosis based on PET-CT and MRI, ultrasonography, physical, or pathological findings. Ascites was detected using ultrasonography, tomographic studies, and intraoperative findings.

Performance status (PS) was measured using ECOG criteria at screening, after combined treatment, and at the end of the study after 5 years of follow-up.

Quality of life (QOL) was assessed at screening, after combined treatment, and at the end of the study after 5 years of follow-up using the European Organization for the Research and Treatment of Cancer Quality of Life Questionnaire-C30, version 3.0. The scales and items of the questionnaire were transformed to a scale of 0–100, using a scoring manual [36]. Evaluation of Global health status, Functional status, and assessment of side effects of the treatment (Symptom scales) was conducted. The Functional scales contained questions about the physical, emotional, cognitive, and social functions. The Symptom scales contained questions about side effects of treatment, such as fatigue, nausea, vomiting, pain, dyspnoea, insomnia, appetite loss, constipation, diarrhoea, and financial difficulties. A high score on a Functional scales or Global health status implies good functioning or high QOL, whereas a high score on a Symptom scales indicates a high degree of complaints or disturbance.

Adverse events were monitored continuously and evaluated using National Cancer Institute Common Terminology Criteria for Adverse Events, version 3.0 [37].

### Statistical analysis

The planned enrollment of 300 patients (*n* = 60 patients per arm) was selected to generate maintenance therapy effect estimates. As a result, 284 patients were enrolled in January 2004 through December 2009 at RSCRR and treated in accordance with their choice made at the moment of diagnosis (arm 1, *n* = 46; arm 2, *n* = 76; arm 3, *n* = 42; arm 4, *n* = 40; arm 5, *n* = 80), according to the modified protocol.

OS and PFS were estimated by Kaplan-Meier analysis [38] and expressed as median value with corresponding 95% confidence interval (95% CI) and 25th, 75th percentiles. Pearson’s correlation coefficients between PFS and OS for all the arms were also calculated.

Multivariate analysis for PFS and OS was performed using Cox proportional hazards model [39]. Hazard ratio (HR), 95% CI, and *p*-value were calculated for the factors likely to influence the survival rate. The PCI cutoff values were determined on the basis of receiver operator characteristic (ROC) curves.

Chi-square test (χ^2^ test) was applied to determine the statistical significance of differences between rates of patients with and without ascites in different arms after combined treatment within 5 years of follow-up, with 95% CIs to be calculated by Klopper-Pearson method. Mann-Whitney U-test, Chi-square test (χ^2^ test), and Student’s t-test were used to estimate the significance of inter-arm differences in other indicators.

Data were analyzed using Statistica package version 10.0 (StatSoft Inc., USA). The Bonferroni correction was used as appropriate to eliminate the multiple comparisons effect. Multivariate analysis was carried out using SPSS statistical software program, version 20.0 (SPSS Inc., Chicago, IL, USA). For all tests, *p*-value < 0.05 was taken as the critical level of significance.

## Results

### Patients

Of 330 eligible screened women with advanced OC, 284 were enrolled between January 2004 and December 2009 at RSCRR and distributed into three maintenance therapy arms and two control arms (arm 1, *n* = 46; arm 2, *n* = 76; arm 3, *n* = 42; arm 4, *n* = 40; arm 5, *n* = 80). Baseline demographic and clinical characteristics were well balanced between treatment arms (Table 1).

Documented CA-125 level - a widely used marker of response in OC trials - at screening was similar among the treatment arms. Further, at presurgery and after completion of combined treatment (after the last course of ACT), СА-125 readings demonstrated a statistically significant benefit to maintenance therapy arms 1–3 compared with control arm 5 (*p* <  0.0001) (Table 1). At presurgery the mean CA-125 level in arm 1 equalled or was less than the threshold CA-125 value (31.50 ± 5.19 U / ml). CA-level in arms 2 and 3 was a little higher than the normal value and was in so-called “grey scale” (37.9 ± 21.43 U / ml and 42.26 ± 24.5 U / ml, respectively) in comparison with control arm 5 without maintenance therapy (68.70 ± 16.23 U / ml (Table 1). CA-125 level in control arm 4 without NACT remained still high (581.98 ± 85.07 U / ml).

Importantly, the vast majority of patients (81–85%) from maintenance therapy arms 1–3 had undergone successful complete cytoreduction as primary debulking surgery, in which all visible tumor foci were removed (*p* <  0.0001). At presurgery moment, patients from arms 1–3 had taken I3C and EGCG agents for 14 days prior to NACT, during NACT, and for a time between the last NACT cycle and surgery. Other patients in arms 1–3 (15–19%) were optimally debulked to ≤ 1 cm. At the same time most patients in control arms 4 and 5 could not be subjected to complete cytoreduction because of lack of technical possibility for such surgery, and so they were optimally debulked to ≤ 1 cm and suboptimally debulked to > 1 cm (Table 1).

### Efficacy

At the time of efficacy analysis, 5 years after combined treatment commencement for the last enrolled patient, 16 patients in arm 1, 28 in arm 2, 11 in arm 3, 19 in arm 4, and 51 in arm 5 had experienced an OS event, while 38 patients in arm 1, 61 in arm 2, 33 in arm 3, 38 in arm 4, and 78 in arm 5 had experienced a PFS event.

**Fig. 2:**
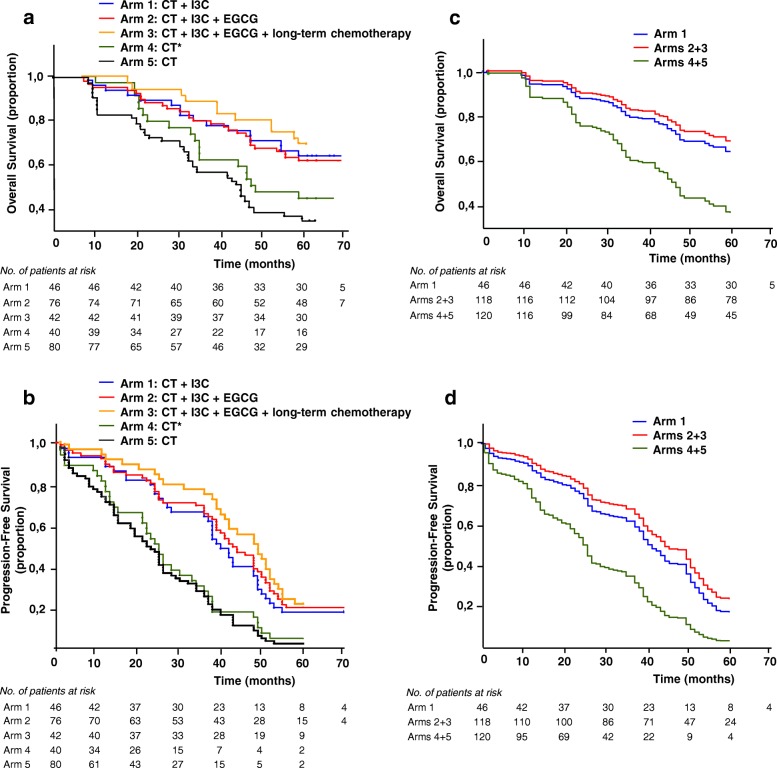
Kaplan–Meier analysis of OS (**a, c**) and PFS (**b, d**) in ovarian cancer patients. Advanced ovarian cancer patients receiving CT plus I3C continuously (arm 1), CT plus I3C and EGCG continuously (arm 2), CT plus I3C and EGCG continuously plus long-term platinum-taxane chemotherapy, 2–3-month cycles (arm 3), CT* alone (arm 4), CT alone (arm 5). *OS* overall survival, *PFS* progression-free survival, *I3C* indole-3-carbinol, *EGCG* epigallocatechin-3-gallate, *CT* combined treatment with neoadjuvant platinum-taxane chemotherapy, CT* combined treatment without neoadjuvant platinum-taxane chemotherapy

Median OS in arms 1–3 was 60.0 months, compared with 46.0 months and 44.0 months in control arms 4 and 5, respectively. Median OS in arms 2+3 (patients receiving I3C and EGCG) was 60.0 months compared with median OS 44.0 months in control arms 4+5 (Fig. 2a, c; Table 2). Median PFS in arm 1 was 39.5 months, in arm 2 – 42.5 months, in arm 3 – 48.5 months, in arm 4 – 24.5 months, in arm 5 – 22.0 months. Median PFS in arms 2+3 was 44.0 months and in control arms 4+5 was 23.0 months (Fig. 2b, d; Table 2).

**Table 2:** Maintenance therapy efficacy analysis

	Arm 1	Arm 2	Arm 3	Arms 2+3	Arm 4	Arm 5	Arms 4+5
	СT + I3C 400 mg	СT + I3C 400 mg + EGCG 200 mg	СT + I3C 400 mg + EGCG 200 mg + long-term chemotherapy	СT + I3C 400 mg + EGCG 200 mg	СT*	СT	СT**
	(*n* = 46)	(*n* = 76)	(*n* = 42)	(*n* = 118)	(*n* = 40)	(*n* = 80)	(*n* = 120)
Primary end point: OS^a^							
Deaths, No. (%) Kaplan-Meier OS time, months	16 (34.8)	28 (36.8)	12 (28.6)	40 (33.9)	24 (60.0)	51 (63.8)	75 (62.5)
Median	60	60	60	60	46	44	44
95% CI	58–60	60–60	60–60	60–60	28–60	33–58	34–54
Q1	47	45	58	47	21.5	22	22
Q3	62	60	60	60	60	60	60
Secondary end point: PFS per RECIST, clinical progression, CA-125 progression, or death^b^							
Kaplan-Meier PFS time, months							
Median	39.5	42.5	48.5	44	24.5	22	23
95% CI	28–49	38–49	39–53	40–49	14–34	15–26	19–26
Q1	24	24.5	36	25	12.5	10.5	11.5
Q3	51	54	55	55	37.5	36.5	37
*r*,	0.811	0.874	0.805	0.855	0.565	0.711	0.661
Secondary end point: Rate of patients with recurrent OC with ascites after combined treatment							
No. (%)	3 (7.9)	5 (8.2)	3 (9.1)	8 (8.5)	24 (63.2)	47 (60.3)	71 (61.2)
95% CI, %	1.7–21.4	2.7–18.1	1.9–24.3	3.7–16.1	46.0–78.2	48.5–71.2	51.7–70.1
Rate of patients with recurrent OC without ascites after combined treatment							
No. (%)	35 (92.1)	56 (91.8)	30 (90.9)	86 (91.5)	14 (36.8)	31 (39.7)	45 (38.8)
95% CI, %	78.6–98.3	81.9–97.3	75.7–98.1	83.9–96.3	21.8–54.0	28.8–51.5	29.9–48.3
*p*^c^	< 0.0001	< 0.0001	< 0.0001		0.7634		
*p*^d^				< 0.0001			

*СT* combined treatment with neoadjuvant chemotherapy, CT* combined treatment without neoadjuvant chemotherapy,

CT** combined treatment with and without neoadjuvant chemotherapy, *I3C* indole-3-carbinol, *EGCG* epigallocatechin-3-gallate,

*OS* overall survival, *PFS* progression-free survival, *95% CI* 95% confidence interval, *Q1* 25th percentile, *Q3* 75th percentile,

*r* Pearson’s correlation coefficient between OS and PFS (*p* < 0.05), *OC* ovarian cancer

^a^Defined as the time from the date of diagnosis to the date of death from any causes. At the time of this analysis, 30 patients in arm 1, 48 patients in arm 2, 31 patients in arm 3, 21 patients in arm 4, 29 patients in arm 5 were censored

^b^Defined as the time from random assignment to disease progression per RECIST, clinical progression (per investigator) or CA-125

progression (per GCIG criteria), or death from any causes. At the time of this analysis, 8 patients in arm 1, 15 patients in arm 2, 9 patients in arm 3, 2 patients in arm 4, 2 patients in arm 5 were censored

^c^Chi-square criterion was applied to determine the differences between arms 1–4 vs arm 5

^d^Chi-square criterion was applied to determine the difference between arms 2+3 vs arms 4+5

Pearson’s correlation coefficients between PFS and OS for arms 1–5 were respectively 0.811, 0.874, 0.805, 0.565 and 0.711, while for arms 2+3 and arms 4+5, respectively, 0.855 and 0.661 *(p <* 0.05) (Table 2).

Multivariate survival analysis performed using Cox proportional hazards model indicated that maintenance therapy with I3C, maintenance therapy with I3C and EGCG, PCI ≤ 25, and FIGO stage III were independent favorable prognostic factors statistically significantly influencing both OS and PFS (*p* <  0.0001 for all above variables) in advanced OC patients. NACT application and secondary debulking surgery at time of relapse did not have prognostic statistical significance both for OS (*p* = 0.246, *p* = 0.930, respectively) and PFS (*p* = 0.521, *p* = 0.205, respectively) (Table 3). Therefore, in this study maintenance therapy with I3C as well as maintenance therapy with I3C and EGCG were the factors significantly associated with survival in advanced OC patients, after adjustment for such variables as neoadjuvant chemotherapy application, initial tumor spread (PCI), FIGO stage III or IV, and secondary surgery at time of relapse.

**Table 3:** Adjusted overall survival and progression-free survival of patients by multivariate Сox regression analyses

Variables	OS		PFS	
	HR (95% CI)	*p*-value	HR (95% CI)	*p*-value
Neoadjuvant chemotherapy	1.367 (0.806–2.319)	0.246	1.136 (0.770–1.678)	0.521
Maintenance therapy with I3C	0.272 (0.147–0.502)	**< 0.0001**	0.309 (0.202–0.472)	**< 0.0001**
Maintenance therapy with I3C and EGCG	0.244 (0.150–0.396)	**< 0.0001**	0.241 (0.172–0.339)	**< 0.0001**
PСI (> 25 vs ≤ 25)	2.829 (1.813–4.415)	**< 0.0001**	2.114 (1.589–2.812)	**< 0.0001**
FIGO stage (IV vs III)	9.642 (5.963–15.592)	**< 0.0001**	6.953 (4.744–10.190)	**< 0.0001**
Secondary debulking surgery	< 0.0001 (< 0.0001- > 1.0х10^3^)	0.930	1.277 (0.875–1.862)	0.205

*OS* overall survival, *PFS* progression-free survival, *HR* hazard ratio, *95% CI* 95% confidence interval, *I3C* indole-3-carbinol, *EGCG* epigallocatechin-3-gallate, *PCI* peritoneal cancer index, *FIGO* International Federation of Gynecology and Obstetrics

NOTE. Bold font indicates *p* < 0.05

After combined treatment within the five-year follow-up period, patients receiving maintenance therapy with I3C as well as maintenance therapy with I3C and EGCG demonstrated a dramatic decrease in ascites OC recurrences: 8 to 9% in arms 1–3 and 8.5% in arms 2+3, vs 60 and 63% in control arms 4 and 5, respectively, and 61% in control arms 4+5 (Table 2). In any comparative combinations, all differences between maintenance therapy arms and control arms were statistically significant (*p <* 0.0001). The total tumor recurrence rate with and without ascites after combined treatment in arms 1–5 was respectively: 82.6%, 80.3%, 78.6%, 95.0%, and 97.5% (Fig. 1, Table 1). The rate of patients without ОС recurrences within 5 years of follow-up in arms 1–5 was respectively: 17.4%, 19.7%, 21.4%, 5.0%, and 2.5% (Table 1).

### Performance status, quality of life and adverse events

ECOG PS score at screening was similar in all the five arms (Table 1), with important statistically significant improvements demonstrated in maintenance therapy arms compared to control in five-year follow-up, 83–88% of surviving patients from arms 1–3 having ECOG scores from 0 to 2, compared to only 55–56% in control arms 4 and 5 (Table 4).

**Table 4:** ECOG performance status of patients after combined treatment and at the end of the study

Characteristic	Arm 1 (*n* = 46)	Arm 2 (*n* = 76)	Arm 3 (*n* = 42)	Arms 2+3 (*n* = 118)	Arm 4 (*n* = 40)	Arm 5 (*n* = 80)
ECOG performance status after combined treatment, No. (%)						
0	35 (76.1)	60 (78.9)	32 (76.2)	92 (77.97)	25 (62.5)	49 (61.25)
1	7 (15.2)	10 (13.2)	6 (14.3)	16 (13.56)	8 (20.0)	15 (18.75)
2	3 (6.5)	4 (5.3)	3 (7.1)	7 (5.93)	4 (10.0)	9 (11.25)
3	1 (2.2)	2 (2.6)	1 (2.4)	3 (2.54)	3 (7.5)	7 (8.75)
p*	0.12	0.0406	0.14	0.0312	0.86	
Alive patients at database cutoff	46 (100)	76 (100)	42 (100)	118 (100)	40 (100)	80 (100)
ECOG performance status at the end of the study, No. (%)						
0	8 (17.4)	15 (19.7)	8 (19.1)	23 (19.49)	2 (5.0)	3 (3.75)
1	9 (19.5)	17 (22.4)	11 (26.2)	28 (23.73)	4 (10.0)	7 (8.75)
2	8 (17.4)	10 (13.2)	7 (16.6)	17 (14.41)	3 (7.5)	6 (7.50)
3	4 (8.7)	4 (5.3)	3 (7.1)	7 (5.93)	4 (10.0)	7 (8.75)
4	1 (2.2)	2 (2.6)	1 (2.4)	3 (2.54)	3 (7.5)	6 (7.50)
p*	0.0180	0.0013	0.0078	0.0007	0.86	
Death	16 (34.8)	28 (36.8)	12 (28.6)	40 (33.90)	24 (60.0)	51 (63.75)
Alive patients at database сutoff	30 (62.5)	48 (63.2)	30 (71.4)	78 (66.10)	16 (40.0)	29 (36.25)

*ECOG* Eastern Cooperative Oncology Group

^*^Mann-Whitney U-test was applied to determine the differences between arms 1–4 vs arm 5 and arms 2+3 vs arm 5

The trend was the same in QOL comparative assessments using the EORTC QLQ-C30 questionnaire. All the results on EORTC QLC-C30 performed at baseline, after combined treatment, and at the end of the study are summarized in Tables 5, 6 and 7. While at screening there was no statistically significant difference in EORTC QLC-C30 scores between maintenance therapy arms and control arm 5, the former demonstrated statistically significant improvements in Global Health Status and Functional Status after 5 years of follow-up. Importantly, arm 3 patients, despite being subject to long-term chemotherapy, reported the same elevated Functional Status as compared to control arm 5 as arm 2 patients who were under the same I3C and EGCG regimen but without the long-term chemotherapy.

**Table 5:** Global health status in maintenance therapy arms 1–3, and arms 2+3 versus control arm 5

Characteristic	Arm 1 (*n* = 46)	Arm 2 (*n* = 76)	Arm 3 (*n* = 42)	Arms 2+3 (*n* = 118)	Arm 5 (*n* = 80)
At screening					
MS ± SD	77.51 ± 10.24	78.83 ± 10.35	76.97 ± 10.96	78.18 ± 10.56	78.24 ± 9.75
p*	0.6733	0.7162	0.5236	0.9690	
After combined treatment					
MS ± SD	63.51 ± 9.64	64.95 ± 8.65	63.52 ± 9.77	64.45 ± 9.04	63.98 ± 7.46
p*	0.7475	0.4603	0.7724	0.7085	
At the end of the study					
MS ± SD	55.37 ± 20.48	63.69 ± 14.88	58.23 ± 18.33	61.57 ± 16.41	53.19 ± 16.52
p*	0.6532	**0.0060**	0.2796	**0.0240**	

*MS* mean score, *SD* standard deviation

*Student’s test was applied to determine the differences between arms 1–3 vs arm 5 and arms 2+3 vs arm 5

NOTE. Bold font indicates *p* < 0.05

**Table 6:** Functional scales in maintenance therapy arms 1–3, and arms 2+3 versus control arm 5

Characteristic	Arm 1 (*n* = 46)	Arm 2 (*n* = 76)	Arm 3 (*n* = 42)	Arms 2+3 (*n* = 118)	Arm 5 (*n* = 80)
At screening					
MS ± SD	81.23 ± 4.42	82.05 ± 4.62	82.18 ± 4.62	82.09 ± 4.60	81.87 ± 5.16
p*	0.4464	0.8218	0.7500	0.7516	
After combined treatment					
MS ± SD	66.49 ± 5.10	67.51 ± 4.78	66.95 ± 4.84	67.32 ± 4.79	67.15 ± 4.92
p*	0.4440	0.6504	0.8337	0.8202	
At the end of the study					
MS ± SD	50.90 ± 9.74	55.04 ± 6.68	53.41 ± 7.39	54.41 ± 6.96	48.10 ± 6.09
p*	0.2122	**< 0.0001**	**0.0055**	**0.0001**	

*MS* mean score, *SD* standard deviation

*Student’s test was applied to determine the differences between arms 1–3 vs arm 5 and arms 2+3 vs arm 5

NOTE. Bold font indicates *p* < 0.05

**Table 7:** Symptom scales in maintenance therapy arms 1–3, and arms 2+3 versus control arm 5

Characteristic	Arm 1 (*n* = 46)	Arm 2 (*n* = 76)	Arm 3 (*n* = 42)	Arms 2 + 3 (*n* = 118)	Arm 5 (*n* = 80)
At screening					
MS ± SD	19.68 ± 8.52	19.19 ± 8.90	18.19 ± 7.74	18.84 ± 8.49	19.71 ± 8.94
p*	0.9868	0.7207	0.3619	0.4953	
After combined treatment					
MS ± SD	31.39 ± 8.20	31.25 ± 7.77	31.66 ± 5.57	31.39 ± 7.05	31.25 ± 6.58
p*	0.9135	0.9999	0.7376	0.8870	
At the end of the study					
MS ± SD	34.59 ± 21.53	29.28 ± 20.33	30.01 ± 23.19	29.57 ± 21.34	34.83 ± 19.21
p*	0.9644	0.2494	0.3970	0.2588	

*MS* mean score, *SD* standard deviation

*Student’s test was applied to determine the differences between arms 1–3 vs arm 5 and arms 2+3 vs arm 5

In this study, the administration of I3C and EGCG did not negatively affected patients’ general condition and did not cause any additional adverse events (AEs) beyond the AEs caused by the administration of standard chemotherapy drugs (Table 8). All reported treatment-related AEs were stopped independently or by symptomatic therapy. There were no AEs requiring reduction in standard chemotherapy dosages or any changes in the regimen of standard and/or maintenance therapy. There were no cases of discontinuing, or changing the recommended dosages of I3C- and EGCG-containing drugs. There were no treatment-related deaths.

**Table 8:** Treatment-related grade ≥ 2 AEs in ≥ 25% of patients in ≥ 1 treatment arms*

Characteristic	Arm 1 (*n* = 46)	Arm 2 (*n* = 76)	Arm 3 (*n* = 42)	Arm 4 (*n* = 40)	Arm 5 (*n* = 80)
Hematologic, No. (%)					
Anemia (grades 1–2)	35 (76.1)	56 (73.7)	32 (76.2)	31 (77.5)	63 (78.8)
Leukopenia	30 (65.2)	51 (67.1)	28 (66.7)	27 (67.5)	52 (65.0)
Neutropenia	34 (73.9)	54 (71.1)	30 (71.4)	29 (72.5)	59 (73.8)
Trombocytopenia	38 (82.6)	62 (81.6)	35 (83.3)	32 (80.0)	65 (81.3)
Constitutional symptoms, No. (%)					
Fatigue	41 (89.1)	67 (88.2)	37 (88.1)	35 (87.5)	69 (86.3)
Insomnia	40 (87.0)	68 (89.5)	35 (83.3)	34 (85.0)	71 (88.8)
Body weight loss	35 (76.1)	58 (76.3)	32 (76.2)	30 (75.0)	62 (77.5)
Increased perspiration	39 (84.8)	61 (80.3)	34 (81.0)	32 (80.0)	65 (81.3)
Dermatologic, No. (%)					
Alopecia (partial or total)	46 (100)	76 (100)	42 (100)	40 (100)	80 (100)
Nail changes	37 (80.4)	61 (80.3)	34 (81.0)	33 (82.5)	65 (81.3)
Rash	14 (30.4)	23 (30.3)	13 (31.0)	13 (32.5)	27 (33.8)
Gastrointestinal, No. (%)					
Decreased appetite	40 (87.0)	67 (88.2)	37 (88.1)	35 (87.5)	71 (88.8)
Constipation/Diarrhea	12 (26.1)	19 (25.0)	12 (28.6)	11 (27.5)	23 (28.8)
Dispepsia	41 (89.1)	67 (88.2)	38 (90.5)	35 (87.5)	73 (91.3)
Nausea/Vomiting	30 (65.2)	51 (67.1)	27 (64.3)	26 (65.0)	53 (66.3)
Abdominal pain	19 (41.3)	31 (40.8)	18 (42.9)	17 (42.5)	35 (43.8)
Metabolic, No. (%)					
Hypomagnesemia	13 (28.3)	22 (28.9)	13 (31.0)	12 (30.0)	25 (31.3)
Hyper/hyponatremia	14 (30.4)	24 (31.6)	12 (28.6)	13 (32.5)	24 (30.0)
Hypocalcemia	11 (23.9)	17 (22.4)	10 (23.8)	9 (22.5)	20 (25.0)
Alkaline phosphatase increased	12 (26.1)	19 (25.0)	10 (23.8)	10 (25.0)	19 (23.8)
Neuromuscular & skeletal, central nervous system, otic, ocular, No. (%)					
Peripheral neuropathy	10 (21.7)	18 (23.7)	8 (19.0)	10 (25.0)	18 (22.5)
Arthralgia/myalgia	28 (60.9)	45 (59.2)	24 (57.1)	23 (57.5)	49 (61.3)
Dizziness	14 (30.4)	24 (31.6)	13 (31.0)	12 (30.0)	26 (32.5)
Memory impairment	35 (76.1)	58 (76.3)	33 (78.6)	31 (77.5)	60 (75.0)
Ototoxicity	14 (30.4)	24 (31.6)	13 (31.0)	12 (30.0)	25 (31.3)
Retinopathy	11 (23.9)	19 (25.0)	9 (21.4)	9 (22.5)	17 (21.3)
Urogenital, No. (%)					
Pain/difficulty urinating	12 (26.1)	18 (23.7)	11 (26.2)	9 (22.5)	19 (23.8)
Others	14 (30.4)	23 (30.3)	14 (33.3)	13 (32.5)	25 (31.3)

*AEs* adverse events

*Chi-square criterion was applied to determine the differences between arms

All differences between maintenance therapy arms 1–3 vs control arms 4 and 5 were statistically insignificant (*p* > 0.2)

## Discussion

Ovarian cancer, also known colloquially among oncologists as the “Silent Killer,” still presents a formidable challenge. This malignancy remains associated with high rates of morbidity and mortality because it is largely asymptomatic in the early stages, which leads to late diagnosis, while the tumor itself is prone to broad early dissemination, active metastasis, and multidrug resistance emerging after chemotherapy.

A great deal of effort is made to improve current conventional treatment of OC. Some interesting promising approaches obtaining encouraging results to manage recurrent OC became known during last years, such as hyperthermic intraperitoneal intraoperative chemotherapy following secondary cytoreduction in recurrent platinum-sensitive OC patients [40, 41]. However, the medical community is not yet in a position to state that, in general, enhancements of conventional treatment methods or combined usage of conventional chemotherapy with modern monotargeted antitumor drugs have translated into tangible and significant progress in improving the outcomes of OC patients [5–8, 42, 43]. Apparently, the main cause of this failure is insufficient and incomprehensive understanding of the cellular and molecular biology of heterogeneous chemoresistant and recurrent ovarian tumors.

In recent years, OC has repeatedly and strongly been described as a cancer stem cell disease [19, 20, 44–47]. Ovarian CSCs were found experimentally in vitro and in vivo, and clinically in OC patients’ primary tumors, ascites and secondary tumor foci [19, 20]. In response to adjuvant chemotherapy with platinum derivatives (in combination with taxanes as well as individually), which effectively eliminated the bulk of ovarian tumor cells, those stem cell phenotype cancer cells not only survived but more than that, proliferated and demonstrated elevated tumorigenic and metastatic activity in vivo [44], even after a short–term single treatment of conventional chemotherapy [48].

It was also established that in all patients who suffered from recurrent and metastatic OC after conventional treatment, the rate of CSCs in recurrent tumors and ascites was dramatically higher than in their primary tumors, therefore, ovarian CSCs can be considered a prognostic factor for OC relapse [22]. There is evidence on CSC rate correlating with recurrence and survival of patients with early OC [21]. It was shown that OC-associated ascites acts as a great pool of CSCs whose number and tumorigenic activity dramatically increased after standard chemotherapy [49].

Recently, an independent concept of ovarian CSCs was formulated as part of the overall CSC framework, with a novel ovarian carcinogenesis model leading to a new OC treatment approach based on a combination of conventional chemotherapy drugs with specific ovarian CSC inhibitors [44]. The newly suggested more effective approach to OC management by drug therapy was to target CSCs using specific CSCs inhibitors and kill bulk tumor cells using standard chemotherapy together. There is every reason to believe that the better we understand the regulation of ovarian CSCs activity, the easier it will be to develop improved therapeutic strategies for recurrent ovarian cancer [50].

To date, multiple anticarcinogenic activities of unique non-toxic natural compounds I3C, its in vivo metabolite 3,3′-diindolylmethane (DIM) (it is considered that all the basic antitumor properties of I3C are due to those of DIM), and EGCG were comprehensively studied in different malignancies and amply described [51–54], including in OC [55–57].

These natural agents have been shown to suppress proliferation of tumor cells, selectively induce their cell cycle arrest and apoptosis. They have also demonstrated anti-angiogenic, anti-migratory, anti-metastatic, anti-inflammatory, and anti-oxidant activity, including at the transcription level. I3C and DIM as ligands of aryl hydrocarbon receptors influence phase-I and II carcinogen and xenobiotic metabolism and normalize abnormal estrogen metabolism by increasing the production of less estrogenic 2-hydroxyestrone and thus improving the 2-hydroxyestrone/16α-hydroxyestrone ratio in blood and estrogen dependent tissues. I3C, DIM, and EGCG also have epigenetic antitumor activity inhibiting the key epigenetic enzymes like DNA methyltransferase [58–61] and histone deacetylase [62–64]. Today EGCG is considered to be one of the most promising anti-cancer agents with DNA-demethylating epigenetic activity comparable to current FDA-approved DNA-demethylating epigenetic drugs [65]. I3C, DIM, and EGCG can also modulate non-coding miRNA expression profiles, leading to the inhibition of cancer cell growth, induction of apoptosis, reversal of epithelial-mesenchymal transition, or enhancement of efficacy of conventional cancer therapeutics [66].

It is very important that I3C [67], DIM [68–70], and EGCG [71–74] were found to be capable of selectively inhibiting CSCs by specifically blocking the key molecular targets responsible for their stemness and chemoresistance, and soluble factors of their proinflammatory niche, determining CSCs tumorigenicity. These targets are: the components of Wnt, Hedgehog, and SHh signaling pathways; Nanog, Lin28B, c-Myc, Oct-4, NF-κB, and STAT3 transcription factors; TGFβ, EGF, bFGF growth factors and their receptors, inducible nitric oxide synthase, pro-inflammatory cytokines, matrix metalloproteinases, pro-angiogenic factors VEGF and HIFα, TLR4 receptors, etc. It was also estimated that I3C, DIM and EGCG inhibit epithelial-mesenchymal transition (EMT) signatures, which correlates with essentially reduced CSC’s migration and invasion, and subsequent metastasis [75, 76]. It is known that the EMT in cancer cells is associated with CSC’s phenotype and their invasive and metastatic activity as well as multidrug resistance.

So there is a broad discussion of possibilities related to using these compounds not only as promising chemopreventive agents, but also as effective adjuvants in combined cancer therapy, enhancing the effect of conventional cancer therapies through additive, synergistic effects and amelioration of deleterious side effects, preventing tumor recurrences, and reducing metastasis. In OC, the capacity of EGCG to enhance cisplatin sensitivity was established [77]. I3C, DIM and EGCG were evaluated in many human phase I and phase II clinical trials as potential chemopreventive agents or chemo-radio-sensitizers in human cancers.

In our trial, oral administration of I3C and EGCG as maintenance therapeutic agents in advanced OC has demonstrated dramatic increase in median OS (almost one and a half times) and median PFS (approximately double). HR levels calculated with multivariate Cox regression analysis indicate that long-term maintenance therapy with I3C as well as maintenance therapy with I3C and EGCG are independent favorable prognostic factors statistically significantly associated with increase in OS and PFS after adjustment for some variables, including PCI, having well-known prognostic value in primary advanced OC [25, 29].

Patients receiving I3C or I3C and EGCG also demonstrated statistically significant dramatic decrease in OC relapses with ascites: 8–9% vs 60–63%. Ascites in OC patients is known to correlate with peritoneal dissemination of OC and unfavorable prognosis [78, 79].

Recent recognition of a fundamental CSCs role in recurrent OC development and the discovery of metastatically active chemoresistant CSCs in ascites has provided additional support, suggesting that ascites facilitates extensive CSCs peritoneal dissemination and emergence of local recurrences and metastatic foci that are resistant to conventional chemotherapy. The hypothesis therefore is that I3C and EGCG, administered as part of maintenance therapy during and after combined treatment, inhibit ovarian CSCs and thereby dramatically decrease ascites relapse incidence, which in turn results in significantly better survival rates and higher median OS and PFS.

Importantly, the benefit of maintenance therapy with I3C as well as I3C and EGCG was first revealed as early as at the presurgery stage. The vast majority of patients (81–85%) in maintenance therapy arms 1–3 could be subjected to successful complete cytoreductive surgery, in which all visible tumor foci were removed. At the same time there was no technical possibility to do so in most patients from control arms 4 and 5. Thus, the vast majority of patients in control arms 4 and 5 could not be subjected to complete cytoreduction, they were optimally debulked to ≤ 1 cm and suboptimally debulked to > 1 cm. The prognostic significance of residual tumor after primary OC surgery has been recognized worldwide, and several scores were developed with a view to better predict it [34, 80]. We have a good reason to believe that the possibility of more radical surgery in most patients from arms 1–3 can be explained by favorable multiple antitumor effect of I3C and EGCG including their anti-CSCs activity established earlier in numerous studies. At presurgery moment, patients from maintenance therapy arms 1–3 had taken I3C and EGCG agents for 14 days prior to NACT, during NACT, and for a time between the last NACT cycle and surgery.

Also, the levels of tumor marker СА-125 were statistically significantly lower in arms 1–3 at presurgery and after combined treatment compared to control (combined treatment alone).

The prolonged administration of I3C and EGCG as maintenance therapeutic agents facilitated a statistically significant improvement in the PS score (Table 4) and key QOL scores as per EORTC QLQ-C 30 (Tables 5 and 6, for references to the prognostic value of QOL and PS see [81–83]).

In addition, I3C and EGCG are safe compounds and have not demonstrated any toxicity. In a previous placebo controlled clinical trial, the level of AEs in the group receiving I3C was not statistically significantly different from that in the placebo group [84].

Our comparative clinical study is the first trial that investigates the efficacy of orally administered I3C and EGCG as long-term maintenance therapeutic agents in advanced ovarian cancer. The results of this study should be regarded as preliminary, and the study itself as hypothesis-generating. More blinded randomized trials on larger samples of OC patients in this treatment regimen are required, with adjustments for as many factors possibly affecting survival and the intrinsic biologic response rate as possible. In addition to PCI, FIGO stage, histological type, and grading, one such factor should be how many NACT cycles the patient needed to achieve an optimal therapeutic efficacy evaluated by CA-125 level dynamics and by tumor response per RECIST at presurgery. In our study, some patients needed only 2 cycles of NACT, while others needed 3 or 4 cycles, and the proportion of “two-cycle” patients was higher in maintenance therapy arms 1–3 (84.8%, 85.5%, 85.7%, respectively) than in control arm 5 (68.8%) (data not shown). Our data nevertheless demonstrated that the suggested and documented maintenance therapy, starting before and continued during the combined treatment and subsequently for 5 years of follow-up, can be considered to be a promising effective and safe approach to increase the efficacy of treatment of advanced ovarian cancer.

## Conclusions

In summary, we showed that the usage of suggested doses of I3C and EGCG as pharmaceutical agents prior to and during combined treatment included neoadjuvant platinum-taxane chemotherapy, surgery, and adjuvant platinum-taxane chemotherapy with their subsequent long-term administration can be considered a new promising way of providing maintenance therapy to advanced ovarian cancer patients [85], which achieved much better treatment outcomes, significantly improved patients’s survival and quality of life. In the future, these results may contribute to the development of more efficacious and safe treatment approaches and regimens in ovarian cancer and other female reproductive malignancies.

## Abbreviations

ACT: Adjuvant chemotherapy
AEs: Adverse events
ALT: Alanine aminotransferase
AST: Aspartate aminotransferase
CI: Confidence interval
CSCs: Cancer stem cells
CT: Combined treatment
DIM: 3,3′-Diindolylmethane
ECOG: Eastern Cooperative Oncology Group
EGCG: Epigallocatechin-3-gallate
FIGO: International Federation of Gynecology and Obstetrics
HR: Hazard ratio
I3C: Indole-3-carbinol
MRI: Magnetic resonance imaging
NACT: Neoadjuvant chemotherapy
OC: Ovarian cancer
OS: Overall survival
PCI: Peritoneal cancer index
PET-CT: Positron emission tomography-computed tomography
PFS: Progression-free survival
PS: Performance status
QOL: Quality of life
RSCRR: Russian Scientific Center of Roentgenoradiology
ЕМТ: Epithelial-mesenchymal transition

## References

[1] Globocan (2012). Cancer incidence and mortality worldwide: IARC Cancer Base No11. Lyon.

[2] Armstrong DK, Bundy B, Wenzel L, Huang HQ, Baergen R, Lele S (2006). Intraperitoneal cisplatin and paclitaxel in ovarian cancer. N Engl J Med.

[3] Katsumata N, Yasuda M, Isonishi S, Takahashi F, Michimae H, Kimura E (2013). Long-term results of dose-dense paclitaxel and carboplatin versus conventional paclitaxel and carboplatin for treatment of advanced epithelial ovarian, fallopian tube, or primary peritoneal cancer (JGOG 3016): a randomised, controlled, open-label trial. Lancet Oncol.

[4] Bookman MA, Brady MF, McGuire WP, Harper PG, Alberts DS, Friedlander M (2009). Evaluation of new platinum-based treatment regimens in advanced-stage ovarian cancer: a phase III trial of the gynecologic Cancer intergroup. J Clin Oncol.

[5] Bookman MA, Darcy KM, Clarke-Pearson D, Boothby RA, Horowitz IR (2003). Evaluation of monoclonal humanized anti-HER2 antibody, trastuzumab, in patients with recurrent or refractory ovarian or primary peritoneal carcinoma with overexpression of HER2: a phase II trial of the gynecologic oncology group. J Clin Oncol.

[6] Noguera IR, Sun CC, Broaddus RR, Branham D, Levenback CF, Ramirez PT (2012). Phase II trial of imatinib mesylate in patients with recurrent platinum and taxane-resistant low-grade serous carcinoma of the ovary, peritoneum, or fallopian tube. Gynecol Oncol.

[7] Sato S, Itamochi H (2012). Bevacizumab and ovarian cancer. Curr Opin Obstet Gynecol.

[8] Oza AM, Cook AD, Pfisterer J, Embleton A, Ledermann JA, Pujade-Lauraine E (2015). Standard chemotherapy with or without bevacizumab for women with newly diagnosed ovarian cancer (ICON7): overall survival results of a phase 3 randomised trial. Lancet Oncol..

[9] Giornelli GH (2016). Management of relapsed ovarian cancer: a review. Springerplus.

[10] du Bois A, Floquet A, Kim JW, Rau J, del Campo JM, Friedlander M (2014). Incorporation of pazopanib in maintenance therapy of ovarian cancer. J Clin Oncol.

[11] Berek J, Taylor P, McGuire W, Smith LM, Schultes B, Nicodemus CF (2009). Oregovomab maintenance monoimmunotherapy does not improve outcomes in advanced ovarian cancer. J Clin Oncol.

[12] Frampton JE (2015). Olaparib: a review of its use as maintenance therapy in patients with ovarian cancer. BioDrugs.

[13] Mirza MR, Monk BJ, Herrstedt J, Oza AM, Mahner S, Redondo A (2016). Niraparib maintenance therapy in platinum-sensitive, recurrent ovarian cancer. N Engl J Med.

[14] Chaffer CL, Weinberg RA (2011). A perspective on cancer cell metastasis. Science.

[15] Croker AK, Allan AL (2008). Cancer stem cells: implications for the progression and treatment of metastatic disease. J Cell Mol Med.

[16] Mimeault М, Batra SK (2010). New promising drug targets in cancer- and metastasis-initiating cells. Drug Discov Today.

[17] Сhen K, Huang YH, Chen JL (2013). Understanding and targeting cancer stem cells: therapeutic implications and challenges. Acta Pharmacol Sin.

[18] Wicha MS, Liu S, Dontu G (2006). Cancer stem cells: an old idea – a paradigm shift. Cancer Res.

[19] Foster R, Buckanovich RJ, Rueda BR (2013). Ovarian cancer stem cells: working towards the root of stemness. Cancer Lett.

[20] Ahmed N, Abubaker K, Findlay J, Quinn M (2013). Cancerous ovarian stem cells: obscure targets for therapy but relevant to chemoresistance. J Cell Biochem.

[21] Steffensen KD, Alvero AB, Yang Y, Waldstrom M, Hui P, Holmberg JC (2011). Prevalence of epithelial ovarian cancer stem cells correlates with recurrence in early-stage ovarian cancer. J Oncol.

[22] Hosonuma S, Kobayashi Y, Kojo S, Wada H, Seino K, Kiguchi K (2011). Clinical significance of side population in ovarian cancer cells. Hum Cell.

[23] Sarkar FH (2010). Current trends in the chemoprevention of cancer. Pharm Res.

[24] Kurman RJ, Сarcanqiu ML, Herrington CS, Young RH (2014). WHO Classificaihion of tumours of female reproductive organs.

[25] Llueca A, Escrig J, MUAPOS working group (multidisciplinary unit of abdominal pelvic oncology surgery) (2018). Prognostic value of peritoneal cancer index in primary advanced ovarian cancer. Eur J Surg Oncol.

[26] Hofmann MA, Hauschild A, Mohr P, Garbe C, Weichenthal M, Trefzer U (2011). Prospective evaluation of supportive care with or without CVD chemotherapy as a second-line treatment in advanced melanoma by patient's choice: a multicentre Dermatologic Cooperative Oncology Group trial. Melanoma Res.

[27] Wright AA, Bohlke K, Armstrong DK, Bookman MA, Cliby WA, Coleman RL (2016). Neoadjuvant chemotherapy for newly diagnosed, advanced ovarian cancer: Society of Gynecologic Oncology and American Society of Clinical Oncology Clinical Practice Guideline. Gynecol Oncol.

[28] Fagotti A, Ferrandina G, Vizzielli G, Fanfani F, Gallotta V, Chiantera V (2016). Phase III randomised clinical trial comparing primary surgery versus neoadjuvant chemotherapy in advanced epithelial ovarian cancer with high tumour load (SCORPION trial): final analysis of peri-operative outcome. Eur J Cancer.

[29] Tentes AG, Tripsiannis G, Markakidis S, Karanikiotis C, Tzegas G, Georgiadis G (2003). Peritoneal cancer index: a prognostic indicator of survival in advanced ovarian cancer. EJSO.

[30] Rustin GJ, Quinn M, Thigpen T, du Bois A, Pujade-Lauraine E, Jakobsen A (2004). Re: new guidelines to evaluate the response to treatment in solid tumors (ovarian cancer). J Natl Cancer Inst.

[31] Therasse P, Arbuck SG, Eisenhauer EA, Wanders J, Kaplan RS, Rubinstein L (2000). New guidelines to evaluate the response to treatment in solid tumors: European Organization for Research and Treatment of Cancer, National Cancer Institute of the United States, National Cancer Institute of Canada. J Natl Cancer Inst.

[32] Kiselev VI. Anti-estrogenic and anti-proliferative agent to treat and prevent female reproductive system diseases Patent (invention) No 2315594, Russian Federation, priority awarded September 01, 2006 (*Киселев ВИ. Антиэстрогенное и антипролиферативное средство для лечения и профилактики заболеваний женской репродуктивной системы. Патент на изобретение РФ №2315594, дата приоритета 01 сентября 2006 год*).

[33] Kiselev VI, Muyzhnek EL. Pharmaceutical composition to prevent metastasis and increase the sensitivity (sensitization) of tumors to chemotherapy Drugs Patent (invention) No 2328282, Russian Federation, priority awarded April 23, 2007 (*Киселев ВИ, Муйжнек ЕЛ. Фармацевтическая композиция для профилактики образования метастазов и повышения чувствительности (сенсибилизации) опухолей к химиотерапевтическим препаратам. Патент на изобретение РФ № 2328282, дата приоритета 23 апреля 2007 год*).

[34] Harter P, du Bois A, Hahmann M, Hasenburg A, Burges A, Loibl S (2006). Surgery in Recurrent Ovarian Cancer: The Arbeitsgemeinschaft Gynaekologische Onkologie (AGO) DESKTOP OVAR Trial. Ann Surg Oncol.

[35] Harter P, Sehouli J, Reuss A, Hasenburg A, Scambia G, Cibula D (2011). Prospective validation study of a predictive score for operability of recurrent ovarian cancer: the multicenter intergroup study DESKTOP II. A project of the AGO Kommission OVAR, AGO study group, NOGGO, AGO-Austria, and MITO. Int J Gynecol Cancer.

[36] Scott NW, Fayers PM (2001). The EORTC QLQ-C30 scoring manual.

[37] Cancer Therapy Evaluation Program: National Cancer Institute Common Terminology Criteria for Adverse Events, version 3.0. http://ctep.cancer.gov. Accessed 9 Aug 2006.

[38] Kaplan EL, Meier P (1958). Nonparametric estimation from incomplete observations. J Am Stat Assoc.

[39] Cox DR (1972). Regression models and life-tables. J R Stat Soc Ser B (Methodological).

[40] Fagotti A, Costantini B, Vizzielli G, Perelli F, Ercoli A, Gallotta V (2011). HIPEC in recurrent ovarian cancer patients: morbidity-related treatment and long-term analysis of clinical outcome. Gynecol Oncol.

[41] Fagotti A, Costantini B, Petrillo M, Vizzielli G, Fanfani F, Margariti PA (2012). Cytoreductive surgery plus HIPEC in platinum-sensitive recurrent ovarian cancer patients: a case-control study on survival in patients with two year follow-up. Gynecol Oncol.

[42] Kaye S. B., Poole C. J., Dańska-Bidzińska A., Gianni L., Del Conte G., Gorbunova V., Novikova E., Strauss A., Moczko M., McNally V. A., Ross G., Vergote I. (2012). A randomized phase II study evaluating the combination of carboplatin-based chemotherapy with pertuzumab versus carboplatin-based therapy alone in patients with relapsed, platinum-sensitive ovarian cancer. Annals of Oncology.

[43] Burger RA, Brady MF, Bookman MA, Fleming GF, Monk BJ, Huang H (2011). Incorporation of bevacizumab in the primary treatment of ovarian cancer. N Engl J Med.

[44] Ahmed N, Abubaker K, Findlay JK (2014). Ovarian cancer stem cells: molecular concepts and relevance as therapeutic targets. Mol Asp Med.

[45] Conic I, Dimov I, Tasic-Dimov D, Djordjevic B, Stefanovic V (2012). Ovarian epithelial cancer stem cells. Sci World J.

[46] Curley MD, Garrett LA, Schorge JO, Foster R, Rueda BR (2011). Evidence for cancer stem cells contributing to the pathogenesis of ovarian cancer. Front Biosci (Landmark Ed).

[47] Djordjevic B, Stojanovic S, Conic I, Jankovic-Velickovic L, Vukomanovic P, Zivadinovic R (2012). Current approach to epithelial ovarian cancer based on the concept of cancer stem cells. J BUON.

[48] Abubaker K, Latifi A, Luwor R, Nazaretian S, Zhu H, Quinn MA (2013). Short-term single treatment of chemotherapy results in the enrichment of ovarian cancer stem cell-like cells leading to an increased tumor burden. Mol Cancer.

[49] Latifi A, Luwor RB, Bilandzic M, Nazaretian S, Stenvers K, Pyman J (2012). Isolation and characterization of tumor cells from the ascites of ovarian cancer patients: molecular phenotype of chemoresistant ovarian tumors. PLoS One.

[50] Kwon MJ, Shin YK (2013). Regulation of ovarian cancer stem cells or tumor-initiating cells. Int J Mol Sci.

[51] Wenga JR, Tsaic CH, Kulp SK, Che CS (2008). Indole-3-carbinol as a chemopreventive and anti-cancer agent. Cancer Lett.

[52] Banerjee S, Kong D, Wang Z, Bao B, Hillman GG, Sarkar FH (2011). Attenuation of multi-targeted proliferation-linked signaling by 3,3′-diindolylmethane (DIM): from bench to clinic. Mutat Res.

[53] Maruthanila VL, Poornima J, Mirunalini S (2014). Attenuation of carcinogenesis and the mechanism underlying by the influence of indole-3-carbinol and its metabolite 3,3′-diindolylmethane: a therapeutic marvel. Adv Pharmacol Sci.

[54] Khan N, Mukhtar H (2008). Multitargeted therapy of cancer by green tea polyphenols. Cancer Lett.

[55] Kandala PK, Srivastava SK (2012). DIMming ovarian cancer growth. Curr Drug Targets.

[56] Trudel D, Labbé DP, Bairati I, Fradet V, Bazinet L, Tetu B (2012). Green tea for ovarian cancer prevention and treatment: a systematic review of the in vitro, in vivo and epidemiological studies. Gynecol Oncol.

[57] Zou M, Zhang X, Xu C (2016). IL6-induced metastasis modulators p-STAT3, MMP-2 and MMP-9 are targets of 3,3′-diindolylmethane in ovarian cancer cells. Cell Oncol (Dordr).

[58] Wu TY, Khor TO, Su ZY, Saw CL, Shu L, Cheung KL (2013). Epigenetic modifications of Nrf2 by 3,3′-diindolylmethane in vitro in TRAMP C1 cell line and *in vivo* TRAMP prostate tumors. The AAPS J.

[59] Haefele A, Word B, Yongmei X, Hammons GJ, Lyn-Cook BD (2007). Indole-3-carbinol (I3C) modulates expression of DNA methyltransferases 1, 3a, and 3b in pancreatic cancer cells: effects of gender and a novel (C→T) polymorphism in the promoter region of DNMT 3b. Int J Cancer Prev.

[60] Fang MZ, Wang Y, Ai N, Hou Z, Sun Y, Lu H (2003). Tea polyphenol (−)-epigallocatechin-3-gallate inhibits DNA methyltransferase and reactivates methylation-silenced genes in cancer cell lines. Cancer Res.

[61] Lyn-Cook BD, Mohammed SI, Davis C, Word B, Haefele A, Wang H (2010). Gender differences in gemcitabine (Gemzar) efficacy in cancer cells: effect of indole-3-carbinol. Anticancer Res.

[62] Li Y, Li X, Guo B (2010). Chemopreventive agent 3,3′-diindolylmethane selectively induces proteasomal degradation of class I histone deacetylases. Cancer Res.

[63] Pandey M, Shukla S, Gupta S (2010). Promoter demethylation and chromatin remodeling by green tea polyphenols leads to re-expression of GSTP1 in human prostate cancer cells. Int J Cancer.

[64] Beaver LM, Yu TW, Sokolowski EI, Williams DE, Dashwood RH, Ho E (2012). 3,3′-Diindolylmethane, but not indole-3-carbinol, inhibits histone deacetylase activity in prostate cancer cells. Toxicol Appl Pharmacol.

[65] Li Y, Tollefsbol TO (2010). Impact on DNA methylation in cancer prevention and therapy by bioactive dietary components. Curr Med Chem.

[66] Li Y, Kong D, Wang Z, Sarkar FH (2010). Regulation of microRNAs by natural agents: an emerging field in chemoprevention and chemotherapy research. Pharm Res.

[67] Tin AS, Park AH, Sundar SN, Firestone GL (2014). Essential role of the cancer stem/progenitor cell marker nucleostemin for indole-3-carbinol anti-proliferative responsiveness in human breast cancer cells. BMC Biol.

[68] Semov A, Iourtchenco L, Liu LF, Li S, Yan X, Xiaoxue S (2012). Diindolylmethane (DIM) selectively inhibits cancer stem cells. Biochem Biophys Res Commun.

[69] Kong D, Sethi S, Li Y, Chen W, Sakr WA, Heath E (2015). Androgen receptor splice variants contribute to prostate cancer aggressiveness through induction of EMT and expression of stem cell marker genes. Prostate.

[70] Chen D, Banerjee S, Cui QC, Kong D, Sarkar FH, Dou QP (2012). Activation of AMP-activated protein kinase by 3,3′-diindolylmethane (DIM) is associated with human prostate cancer cell death in vitro and in vivo. PLoS One.

[71] Lin СН, Shen YA, Hung PH, Yu YB, Chen YJ (2012). Epigallocathechin gallate, polyphenol present in green tea, inhibits stem-like characteristics and epithelial-mesenchymal transition in nasopharyngeal cancer cell lines. BMC Complement Altern Med..

[72] Li Y, Wicha MS, Schwartz SJ, Suna D (2011). Implications of cancer stem cell theory for cancer chemoprevention by natural dietary compounds. J Nutr Biochem.

[73] Nishimura N, Hartomo TB, Pham TV, Lee MJ, Yamamoto T, Morikawa S (2012). Epigallocatechin gallate inhibits sphere formation of neuroblastoma BE(2)-C cells. Environ Health Prev Med.

[74] Tang SN, Fu J, Nall D, Rodova M, Shankar S, Srivastava RK (2012). Inhibition of sonic hedgehog pathway and pluripotency maintaining factors regulate human pancreatic cancer stem cell characteristics. Int J Cancer.

[75] Ho JN, Jun W, Choue R, Lee J (2013). I3C and ICZ inhibit migration by suppressing the EMT process and FAK expression in breast cancer cells. Mol Med Rep.

[76] Sarkar FH, Li Y, Wang Z, Kong D (2010). The role of nutraceuticals in the regulation of Wnt and Hedgehog signaling in cancer. Cancer Metastasis Rev.

[77] Wang X, Jiang P, Wang P, Yang CS, Wang X, Feng Q (2015). EGCG enhances cisplatin sensitivity by regulating expression of the copper and cisplatin influx transporter CTR1 in ovary cancer. PLoS One.

[78] Tan DS, Agarwal R, Kaye SB (2006). Mechanisms of transcoelomic metastasis in ovarian cancer. Lancet Oncol..

[79] Carmignani CP, Sugarbaker TA, Bromley CM, Sugarbaker PH (2003). Intraperitoneal cancer dissemination: mechanisms of the patterns of spread. Cancer Metastasis Rev.

[80] Friedlander M, Trimble E, Tinker A, Alberts D, Avall-Lundqvist E, Brady M (2011). Clinical trials in recurrent ovarian cancer. Int J Gynecol Cancer.

[81] Coates A, Porzsolt F, Osoba D (1997). Quality of life in oncology practice: prognostic value of EORTC QLQ-C30 scores in patients with advanced malignancy. Eur J Cancer.

[82] Eton D, Fairclough D, Cella D, Yount SE, Bonomi P, Johnson DH (2003). Early change in patient reported health during lung cancer chemotherapy predicts clinical outcomes beyond those predicted by baseline report: results from Eastern Cooperative Oncology Group Study 5592. J Clin Oncol.

[83] Dancey J, Zee B, Osoba D, Whitehead M, Lu F, Kaizer L (1997). Quality of life scores: an independent prognostic variable in a general population of cancer patients receiving chemotherapy. The National Cancer Institute of Canada Clinical Trials Group. Qual Life Res.

[84] Kiselev VI, Smetnik VP, Suturina LV, Selivanov SP, Rudakova EB, Rakhmatullina IR, et al. Indole carbinol (Indinol Forto) is a multitargeted therapy option for cyclic mastodynia. Obstetrics and Gynecology (Russia). 2013; N7:56*–*62 *(Киселев ВИ, Сметник ВП, Сутурина ЛВ, Селиванов СП, Рудакова ЕБ, Рахматуллина ИР и др. Индолкарбинол (Индинол Форто) – метод мультитаргетной терапии при циклической мастодинии. Акушерство и гинекология.* 2013*; N7:56–62)*.

[85] Ashrafyan LA, Kiselev VI, Paltsev MA, Kuznetsov IN, Muizhnek EL, Antonova IB, et al. Method to treat ovarian cancer, variants of its metastasis and recurrence. Patent (invention) No 2582939, Russian Federation, priority awarded June 06, 2015 (*Ашрафян ЛА, Киселев ВИ, Пальцев МА, Кузнецов ИН, Муйжнек ЕЛ, Антонова ИБ и др. Способ лечения рака яичников, вариантов его метастазирования и рецидивирования. Патент на изобретение РФ №2582939, дата приоритета 06 июня* 2015 *года*).

